# Effects of Age and Gender in Emotion Regulation of Children and Adolescents

**DOI:** 10.3389/fpsyg.2020.00946

**Published:** 2020-05-26

**Authors:** Alejandro Sanchis-Sanchis, Ma Dolores Grau, Adoración-Reyes Moliner, Catalina Patricia Morales-Murillo

**Affiliations:** ^1^Escuela de Doctorado, Catholic University of Valencia “San Vicente Mártir”, Valencia, Spain; ^2^Faculty of Psychology, Catholic University of Valencia “San Vicente Mártir”, Valencia, Spain

**Keywords:** emotion regulation, emotion expression, emotional development, age, gender difference, adolescents

## Abstract

Emotional regulation, understood as the skills and strategies needed to influence and/or modify the emotional experiences, has a very remarkable implication within numerous emotional and behavioral disorders in childhood and adolescence. In recent years there has been a significant increase in research on emotional regulation, however, the results are still divergent in terms of differences in emotional regulation in relation to age and gender. This study aimed to assess emotional regulation in adolescents in relation to their age and gender. Two hundred and fifty-four adolescents from eight schools in the Valencian Community and aged between 9 and 16 years participated in the study. The adolescents completed the Cognitive Emotion Regulation Questionnaire and the FEEL-KJ questionnaire. We analyzed the differences in emotional regulation strategies and a latent emotional regulation variable in two age groups (9–12 years and 13–16 years) and by gender. The results suggested that children and pre-adolescents in the 9–12 year group obtained lower scores in the emotional regulation strategies than the 13–16 year group. Girls reported higher scores on the use of emotional regulation strategies when experiencing sadness, anxiety and anger than boys, and on the overall average of regulation according to these specific emotions. Age, but not gender, had a major effect on scores for the latent variable of emotion regulation. An interaction effect between age and gender was identified in the latent emotion regulation scores. Girls tended to have higher scores than boys when they were younger and lower scores than boys when they were older. These results could be relevant for designing prevention and intervention programs for adolescents and at different ages.

## Introduction

Emotions are an essential part of human functioning, whose aim is to fulfill an adaptive function (Lang et al., 1998; Damasio, 2000) as they give us information about ourselves and the environment around us and predispose us to act accordingly. However, it is necessary to be able to employ processes of emotional regulation in situations where the person requires an adjustment of the emotional response of high intensity. Research on emotional variables and emotional regulation is one of the fastest growing topics since the 1990s (Southam-gerow and Kendall, 2002; Koole, 2009; Tamir, 2011).

One of the most widely used definitions of emotional regulation is proposed by Gross (2015) which refers to “all those processes through which people influence the emotions they have, when they have them and how they experience and express them.” Thompson (1994) defines emotional regulation as “the external and internal processes responsible for monitoring, evaluating and modifying our emotional reactions to meet our goals.” Emotional regulation is generally understood as an adaptive process; however, attempts to regulate our emotions are not always effective.

Differences in regulation and emotional expression according to gender have been a topic of much study in scientific literature. Emotional expression, a skill associated with the regulation of emotions, as pointed out by Gross (1998), is the ability to communicate emotions through their external manifestation, whether verbal or behavioral (Savage et al., 2016).

Emotion regulatory processes involve three mechanisms: input regulation, reappraisal and output regulation (i.e. strategies used to regulate emotional responses including expression of emotion) (Gross, 2001; Schulz and Lazarus, 2012). In this sense, studies on emotional expression collect different theories regarding the why of these gender differences. Firstly, *biological theories* between men and women point out that differences in emotional regulation are due to innate genetic differences or differences that develop with age, such as hormonal differences that appear throughout the maturation period. In this sense, it is pointed out that boys have higher levels of arousal than girls in infancy and early childhood, and on the other hand, they also present a lower capacity for language and inhibitory control (Zahn-Waxler et al., 2008).

Other theories emphasize the importance of the cultural patterns of education received. *Social development theories* point out that children learn behaviors consistent with gender roles, and develop cognitive patterns based on their experience and observation of the environment (Martin and Halverson, 1981; Liben and Bigler, 2002). Children learn both explicitly and through modeling to behave according to gender roles (Bandura, 1969).

Finally, *social constructivist theories* point out the importance of the expression of gender differences based on biological and social differences in development, but also point out the importance of context in the expression of internalized behavior. The expectations of society in general, mark the expression of men’s and women’s behavior according to the gender expectations that the social context has (Deaux and Major, 1987).

In general, studies on differences in the expression of emotions in boys and girls indicate that boys are more likely to present externalizing expressions of emotions while girls are more likely to internalize them. Gender differences in emotional expression are the result of a combination of biologically based temperamental predispositions and on the other hand, the socialization of boys and girls will adopt gender related rules for the expression of emotions (Brody and Hall, 2008).

In this regard, a meta-analysis by Chaplin and Aldao (2013) shows significant, though small, differences in gender roles in the expression of emotions. Thus, girls express more positive emotions and more negative internalizing emotions such as sadness and anxiety, and boys express more externalizing emotions such as anger.

The factors that modulate these gender differences are age, interpersonal context and the type of task in which they are observed (Chaplin and Aldao, 2013). This suggests that gender differences in the expression of emotions may not be static and fixed features in individuals, but are mediated by complex interactions with the environment. The context in which the child develops allows for flexible modulation of emotions, which is a protective factor against the development of psychopathology (e.g. Cole et al., 1994; Bonanno et al., 2004; Cole et al., 2004; Westphal et al., 2010).

Focusing on the use of specific emotional regulation strategies, studies show that girls use more adaptive strategies, such as re-evaluation or active coping, and maladaptive ones, such as rumination and suppression (Chaplin and Aldao, 2013). Adaptive strategies are weakly related to depressive symptoms, while maladaptive strategies are strongly related to depressive symptoms (Orgilés et al., 2018).

This suggests that, although women may use more adaptive strategies than men, this does not help prevent the development of emotional problems. Instead, women’s use of maladaptive strategies, to a greater extent than men, predisposes them to a higher risk for the development of emotional disorders (Chaplin and Aldao, 2013). This is in line with studies that indicate that women have a higher prevalence of emotional disturbance than men (DSM-5; American Psychiatric Association, 2013) and may reflect a more general tendency for women to be more aware of and willing to engage with their emotions than men (Fujita et al., 1991; Nolen-Hoeksema et al., 1999).

With regard to age and emotional regulation, studies show that during childhood and adolescence, the acquisition of skills to modulate emotional responses is closely related to the neurobiological maturation that shapes different levels of organization at the physiological, cognitive and behavioral levels. Similarly, certain characteristics of the context in which children and adolescents develop can favor or hinder the skills with which each learns to express emotions (Campos et al., 2004; Goldsmith and Davidson, 2004; Lewis and Stieben, 2004; Steinberg, 2005; Zeman et al., 2006; Thompson and Goodvin, 2007; Thompson et al., 2008; Luna et al., 2010; Thompson and Goodman, 2010; Thompson, 2011; Cole, 2014).

The interest in the study of emotional variables in child psychopathology has been growing in recent years (Southam-gerow and Kendall, 2002) and has been mainly focused on the study of the emotional regulation strategies that adolescents develop according to the contexts, situations and factors involved.

The coping repertoires of children and adolescents increase with age. As children grow older, instrumental action is complemented by planned problem-solving, they are more capable of attending to and reflecting on their own internal emotional states, and they are increasingly dependent on more sophisticated strategies for coping with emotions (Zimmer-Gembeck and Skinner, 2011).

The regulation of emotions through cognition is an essential part of people’s management of emotions in the face of stressful events (Garnefski et al., 2007). From an early age, children face many stressful events and learn to cope with these situations, in very simple ways and with external strategies, and they increase their repertoire of strategies as they grow up, going from external to internal, i.e. cognitive, strategies (Aldwin, 1994; Fields and Prinz, 1997; Stegge et al., 2004). By the age of eight or nine, children have already learned to regulate their emotions through cognitions or thoughts about themselves, their feelings or others (Harris, 1989; Terwogt and Stegge, 1995; Saarni, 1999).

Studies on the development of cognitive processes, and of brain structures related to the regulation of emotions, suggest that as adolescents mature, they develop more effective regulation of emotions so that they use more adaptive strategies such as the cognitive re-evaluation strategy. However, there is also an increase in the maladaptive *rumination strategy* and less use of the adaptive distraction strategy (Zimmer-Gembeck and Skinner, 2011; Theurel and Gentaz, 2018).

Other authors (Zimmermann and Iwanski, 2014) nuance this change with age, so they find that at age 13–15 adolescents show fewer adaptive regulatory strategies overall than those at age 11 or over 17. They find that in mid-adolescence (13–15 years), adolescents often show a decline in the use of strategies compared to early or late adolescence. This could be explained by the emotional difficulties and conflict with parents reported at this stage (Laursen et al., 1998).

Along the same lines, other research has found that in mid-adolescence there is less use of suppression and cognitive reevaluation compared to younger age groups (Gullone et al., 2010). It was also shown that cognitive reevaluation was negatively related and avoidance positively related to concurrent emotional and behavioral problems in adolescence. Suppression and avoidance were also found to be associated with an increase in emotional and behavioral problems (Flouri and Mavroveli, 2013; Verzeletti et al., 2016).

Emotional regulation has traditionally been considered in the literature as something linked to psychopathology, although as Gross (1998) points out, emotional regulation can be both adaptive and maladaptive, depending on the person, the emotion, its intensity and the context (Bonanno et al., 2004).

There is a growing body of research that points to difficulties in emotional regulation at the base of different psychological disorders and health problems (Fernandez-Berrocal and Extremera, 2014). In this regard, given the high prevalence rates of anxiety disorders in children and adolescents (between 15 and 20%) (Beesdo et al., 2009) makes it especially important to consider emotional regulation in studies of this stage.

If emotion regulation is a risk factor for future psychopathology, techniques aimed at developing adaptive emotion regulation skills should be incorporated into both treatment and preventive interventions. Psychopathology is increasingly considering a transdiagnostic orientation, that is, studying and deepening the basic processes, dimensions, mechanisms, etc. that underlie, maintain and explain the psychopathology of different disorders and diagnostic categories in adults (Fairburn et al., 2003; Barlow et al., 2004; Harvey et al., 2004). In this sense, several meta-analyses (Ewing et al., 2015; García-Escalera et al., 2016) support the effectiveness of the treatment for emotional disorders in adults, children and adolescents (anxiety and depression).

In ligth of the above, we plan to analyze these three questions:

1. What are the emotion regulation strategies used the least and the most by the children and youth in our sample?
2. Are there differences in the mean scores of children and youth in the CERQ and FEEL-KJ emotion regulation strategies by gender and age group?
3. Are there differences in the mean scores of children and youth in the three FEEL-KJ emotions (i.e. anger, sadness, and anxiety) by gender and age group?
4. Are there main and interaction effects of gender and age group on the Emotion Regulation Latent scores of children and youth?

## Materials and Methods

### Participants

Two-hundred and fifty-four children and adolescents, recruited from eight schools in the Valencian Community (i.e. Spanish State), participated in the study. Participants’ ages ranged from 9 to 16 years old. Children and adolescents were classified in one of two groups based on their age and according to the classification of adolescence periods of the World Health Organization (WHO, 2003). The first group consisted of children from 9 to 12 years old (*n* = 118), and the second group consisted of adolescents from 13 to 16 years old (*n* = 115). One-hundred and nine were boys and 102 were girls; data on gender were missing for 43 participants.

Criteria for inclusion of the participant students in the study were to be attending between 4th grade of elementary school (i.e. students turning 10 years old during the school year) and 4th grade of secondary school (i.e. students turning 16 years old during this school year) and having their parents or legal guardian signature in the informed consent form.

The exclusion criterion for children was having an established diagnosis of intellectual disability.

### Instruments

FEEL-KJ: Vragenlijst over emotieregulatie bij kinderen en jongeren [Questionnaire to Assess Children’s and Adolescents Emotion Regulation Strategies Grob and Smolenski, 2005; Dutch version by Braet et al., 2014] is an instrument whose purpose is to measure emotional regulation in different emotions. In addition, the FEEL-KJ measures both internal and external regulation strategies. Internal regulation includes processes such as directing attention to other stimuli, re-evaluating emotionally charged events, and re-evaluating internal stimuli. External regulation refers to the search for different forms of processing in interaction with the environment, for example, seeking social support or expressing one’s emotions.

It is a self-report measure composed of 90 items that evaluate 15 emotional regulation strategies in response to three basic emotions, anxiety, sadness and anger. Each strategy is evaluated by two items on a Likert scale (from 1 = rarely to 5 = almost always). The instrument consists of 15 primary scales (average of the three emotions together for each regulation strategy) and three secondary scales: a scale of 7 adaptive strategies (acceptance, problem-oriented action, cognitive problem solving, distraction, neglect, reassessment and use of sense of humor), another of 5 maladaptive strategies (giving up, aggressive action, withdrawal, self-assessment and perseverance) and another of 3 additional external regulation strategies (social support, expression and control over the emotion) (Table 1). The FEEL-KJ in its Dutch version has adequate psychometric properties, with an internal consistency (Cronbach’s alpha) of the 15 scales ranging from a α = 0.59 to a α = 0.91. For the secondary scales, α = 0.94 (8–12) and α = 0.95 (13–18) for adaptive strategies; α = 0.89 (8–12) and α = 0.92 for maladaptive strategies and α = 0.79 (8–12) and α = 0.89 (13–18) for external strategies (Braet et al., 2014).

**TABLE 1:** Emotion regulation strategies of the FEEL-KJ.

Emotion regulation strategies	Example of the strategies
**Adaptive strategies**	
Problem solving	“I try to change what makes me angry”
Distraction	“I do something fun”
Forgetting	“I think it will pass”
Acceptance	“I accept what makes me angry”
Humor enhancement	“I think about things that make me happy”
Cognitive problem solving	“I think about what I can do”
Revaluation	“I tell myself it is nothing important”
**Maladaptive strategies**	
Giving up	“I don’t want to do anything”
Withdrawal	“I don’t want to see anyone”
Rumination	“I cannot get it out of my head”
Self-devaluation	“I blame myself”
Aggressive actions	“I get into a quarrel with others”
**External strategies**	
Social support	“I tell someone how I am doing”
Expression	“I express my anger”
Emotional Control	“I keep my feelings for myself”

*Adapted from Grob and Smolenski (2005).*

The internal consistency index (i.e. Cronbach’sα) for the scores in our study by strategy, emotion, and factor are shown in Table 2. The internal consistency of the Disadaptive and Adaptive Emotion strategies was 0.83 and 0.94, respectively. For external regulation strategies it was 0.85. In addition to Cronbach Alpha, a more suitable test for internal consistency of ordinal scales was used (i.e. Ordinal Alpha or *KR-20*). Ordinal Alpha values of the FEEL-KJ scores indicated 12 out of the 15 FEEL-KJ strategies had internal consistency values above 0.70, ranging from 0.61 to 0.98 (Table 2).

**TABLE 2:** Descriptive Statistics for the FEEL-KJ and CERQ Participants’ Overall Scores.

Subscales and dimensions	Min – Max	*M (SD)*	*Skewness*	*Kurtosis*	*Cronbach Alpha (α)*	*Ordinal Alpha (KR-20)*
**FEEL-KJ (*N* = 254)**						
**Factors**						
Adaptive	40–207	136.09(28.2)	−0.2(0.15)	−0.19(0.3)	0.94	0.96
Maladaptive	24–130	75.66(16.22)	0.23(0.15)	0.54(0.3)	0.83	0.97
External Regulation	18–90	52.32(13.72)	−0.01(0.15)	−0.14(0.3)	0.85	0.96
**Emotions**						
Anger	44–121	87.22(12.21)	−0.23(0.15)	0.64(0.3)	0.68	0.96
Sadness	46–121	87.92(13.35)	−0.3(0.15)	0.51(0.31)	0.75	0.98
Anxiety	20–131	88.11(13.77)	−0.41(0.15)	2.08(0.3)	0.73	0.98
**Strategies**						
Problem solving	6–30	20.92(4.79)	−0.45(0.15)	−0.11(0.3)	0.74	0.96
Distraction	6–30	20.83(6.32)	−0.28(0.15)	−0.91(0.3)	0.86	0.91
Forgetting	6–30	20.81(6.2)	−0.34(0.15)	−0.74(0.3)	0.85	0.64
Acceptance	4–30	17.32(4.61)	0.08(0.15)	0.05(0.3)	0.64	0.77
Humor Enhancement	4–30	19.69(4.8)	−0.18(0.15)	0.03(0.3)	0.65	0.92
Cognitive Problem Solving	6–30	20.57(5.18)	−0.36(0.15)	−0.18(0.3)	0.80	0.61
Revaluation	4–30	15.96(5)	0.26(0.15)	−0.08(0.3)	0.76	0.86
Giving Up	5–30	14.72(4.73)	0.42(0.15)	0.08(0.3)	0.67	0.81
Withdrawal	4–30	13.24(5.61)	0.62(0.15)	−0.17(0.3)	0.80	0.94
Rumination	5–30	18.22(4.82)	0.1(0.15)	−0.16(0.3)	0.60	0.70
Self-Devaluation	6–30	17.31(4.37)	0.09(0.15)	−0.3(0.3)	0.58	0.85
Aggressive Actions	4–27	12.17(4.72)	0.66(0.15)	−0.16(0.3)	0.75	0.98
Expression	2–34	14.79(5.88)	0.56(0.15)	0.07(0.3)	0.75	0.92
Social Support	6–30	18.61(6.62)	−0.08(0.15)	−0.9(0.3)	0.88	0.65
Emotional Control	5–30	16.87(5.38)	0.11(0.15)	−0.34(0.3)	0.69	0.80
**CERQ^a^ (*N* = 247)**						
**Factors**						
Maladaptive	17–66	40.68(9.67)	−0.08(0.15)	−0.38(0.31)	0.80	0.91
Adaptive	30–93	62.16(12.06)	0.03(0.15)	−0.26(0.31)	0.82	0.97
**Strategies**						
Self-Blame	4–19	9.74(3.4)	0.57(0.15)	−0.09(0.31)	0.69	0.94
Acceptance	4–19	11.29(3.39)	0.04(0.15)	−0.67(0.31)	0.58	0.94
Rumination	4–20	12.46(3.72)	0.14(0.15)	−0.55(0.31)	0.73	0.91
Positive Refocusing	4–20	12.28(4.55)	0.04(0.15)	−0.91(0.31)	0.84	0.72
Refocus on Planning	4–20	13.56(3.7)	−0.31(0.15)	−0.44(0.31)	0.77	0.77
Positive Reappraisal	4–20	12.21(3.69)	0.08(0.15)	−0.65(0.31)	0.69	0.83
Putting into Perspective	4–20	12.81(3.63)	−0.003(0.15)	−0.38(0.31)	0.64	0.83
Catastrophizing	4–19	9.98(3.57)	0.39(0.15)	−0.43(0.31)	0.68	0.96
Other-Blame	4–20	8.5(3.31)	0.53(0.15)	−0.05(0.31)	0.74	0.73
Total enclosure	52–151	102.84(16.44)	0.53(0.15)	0.15(0.31)	0.82	0.97

*^a^The scores of CERQ and CERQ-K were treated as one variable (CERQ).*

The FEEL-KJ is a tool that can be used both to explore the use of adaptive, maladaptive and external strategies for each individual emotion (anger, anxiety and sadness), and to determine a child or adolescent’s strengths and weaknesses by strategy. Therefore, the goal is not only to identify deficits, but also competencies. The FEEL-KJ can be a valuable tool in studies on the prevalence of adaptive and maladaptive emotional regulation strategies in normal and clinical populations (Aldao et al., 2010).

The FEEL-KJ has been used in different research on emotional regulation and binge eating in children and adolescents from 8 to 13 years old (Czaja et al., 2009), loss of control eating in adolescents from 12 to 18 years old (Goossens et al., 2016) influence of emotional regulation on emotional disorders (Braet et al., 2014; Van Beveren et al., 2016) or the development of emotional regulation throughout childhood and adolescence (Cracco et al., 2017). It’s use would also be useful to study the use of different emotional regulation strategies among different ages as well gender differences, an aspect that we are concerned with in this research.

The *Cognitive Emotion Regulation Questionnaire* (CERQ) (Garnefski et al., 2001) (Spanish version: Dominguez-Sanchez et al., 2013). This instrument evaluates the thoughts that arise from experiencing negative events. It measures the cognitive strategies of emotional regulation that people use when faced with negative or stressful situations in their lives. It is composed of nine subscales: self-blame, acceptance, rumination, positive reorientation, planning, positive re-evaluation, putting oneself in perspective, catastrophism, and blaming others. These strategies are grouped into two categories: adaptive and maladaptive strategies. The adaptive strategies: acceptance, positive refocusing, refocus on planning, positive reappraisal and putting into perspective. The maladaptive strategies: Self-blame, rumination, other-blame and catastrophizing (Table 3). This questionnaire can be applied from the age of 12, both in clinical and non-clinical populations, individually or collectively. It consists of 36 items and each dimension is composed of 4 items. It is a Likert type response questionnaire (from 1 = “almost never”; to 5 = “almost always”). The higher the score in a dimension, the greater the use of this cognitive strategy of emotional regulation. The psychometric properties shown by the instrument in both its original and Spanish versions are satisfactory, the latter obtaining an internal consistency with a Cronbach’s alpha from 0.61 to 0.89.

**TABLE 3:** Cognitive strategies of the CERQ.

Cognitve strategies	Description of the strategies
**Adaptive strategies**	
Putting into perspective	Decrease and relativize the severity of the event
Acceptance	To resign and accept the irreversibility of the negative experience
Positive reappraisal	Thoughts that highlight some positive aspect of the unpleasant event
Positive refocusing	Having pleasent and joyous thougths different from the negative event
Refocus on planning	Thinking about how to solve the problem
**Maladaptive strategies**	
Rumiation	State of excessive worry by negative thoughts and feelings
Catastrophizing	To think about the horrible thing of what happend and conclude that it is the worse experience lived, even compared with what other people have experienced
Self-blame	Thoughts that attribute the cause of the negative event and emotion to oneself
Other-blame	Make others responsible for the negative event that happened

*Adapted from Dominguez-Sanchez et al. (2013).*

The *Cognitive Emotion Regulation Questionnaire – Kids* (CERQ-k) (Garnefski et al., 2007) (English Version: Orgilés et al., 2018). This is the children’s version of the CERQ questionnaire (from 9 to 11 years old) for both clinical and non-clinical populations. It evaluates the thoughts that the child usually has after experiencing a negative event. Like the adult version, it assesses the same nine coping processes with a 36-item Likert type scale (from 1 = “almost never”; to 5 = “almost always”). The psychometric properties show an adequate internal consistency, with Cronbach’s alpha values ranging from 0.67 to 0.79.

The internal consistency indices for the scores in our study for adolescents in the CERQ and CERQ-K and the different subscales are as follows: The internal consistency (α Cronbach’s) of the scores was 0.82 for overall CERQ, 0.82 for adaptive emotional regulation strategies and 0.80 for maladaptive ones. In terms of CERQ dimensions, the α-values ranged from 0.58 to 0.84 (Table 2). When considering Ordinal Alpha values, none of the scores in the subscales, strategies, or the total were below 0.70 (Table 2). Therefore, the internal consistency of these scores was supported.

The CERQ is a widely used instrument in the research of emotional regulation due to its focus on cognitive regulatory strategies (e.g. Garnefski et al., 2002; Zlomke and Hahn, 2010).

Other studies using CERQ analyze how children increase strategies as they grow, learn to regulate emotions, and use more cognitive strategies (Harris, 1989; Aldwin, 1994; Terwogt and Stegge, 1995; Fields and Prinz, 1997; Saarni, 1999; Stegge et al., 2004).

Therefore, the CERQ is a very interesting instrument to evaluate a set of emotional regulation strategies that are being developed in the different ages of the present study (9 to 16 years old). Furthermore, the CERQ allows a distinction to be made between adaptive and maladaptive strategies and is therefore very useful for comparison with the results of the FEEL-KJ, which also establishes this distinction between the strategies evaluated.

To our knowledge, this is the first study that uses the FEEL-KJ with a Spanish sample. Therefore, before data collection started, all scale items were translated into Spanish using translation (Dutch–Spanish) and back-translation (Spanish–Dutch; Hambleton and Li, 2005; Muñiz et al., 2013). For this purpose, a native Dutch speaker, who was fluent in both languages and knowledgeable of both cultures (Dutch and Spanish), translated the original Dutch version into Spanish. Once the scale was translated into Spanish, a different person with the same characteristics as the first translator back-translated it into Dutch. Finally, the original Dutch version of the scale and the back-translated Dutch version were compared to ensure that the original meaning of the items was retained. No cultural adaptations were made to the original version for this study.

To evaluate the validity of the translated version of the FEEL-KJ to measure the emotion regulation construct in our study, the scores of the participants in the FEEL-KJ factors have been correlated with the scores in the CERQ dimensions. The CERQ has been validated in the Spanish population (Dominguez-Sanchez et al., 2013; Orgilés et al., 2018), therefore statistically significant correlations among the scores of the FEEL-KJ factors and the scores of the CERQ dimensions in our sample would contribute to support the validity of the FEEL-KJ to measure emotion regulation. The scores in the FEEL-KJ factor of Adaptive Strategies correlated in a positively, statistically significant and moderately manner with the CERQ Adaptive dimension, *r* = 0.60, *p* < 0.01. As for the FEEL-KJ Maladaptive Strategies factor, the scores were positively, moderately, and statistically significantly related to the CERQ Maladaptive dimension scores, *r* = 0.50, *p* < 0.01. No correlations were identified among the scores of the FEEL-KJ External Regulation Factor and the CERQ Adaptive (*r* = 0.09, *p* > 0.05) and Maladaptive (*r* = −0.01, *p* > 0.05) dimensions scores.

### Procedure

This study is part of a bigger project regarding the emotion regulation of children and youth living in the Valencian Autonomous Community, Spain. After an Institutional Review Board approved the project, we contacted the principles of the schools to request permission to invite students to participate in the study. Parents signed an informed consent, and we met with the leaders of each school to coordinate the most appropriate schedules for evaluating the participants.

The study was conducted in accordance with the Declaration of Helsinki. Approval was obtained from the Institutional Review Board of the Catholic University of Valencia “San Vicente Mártir”, Spain.

### Data Analysis

The statistical package SPSS 21 (IBM Corp, 2012) was used to run descriptive statistics, correlational analyses, univariate analyses, and multivariate analyses to describe our sample’s scores on the FEEL-KJ and CERQ questionnaires and evaluate main and interaction effects among the studied variables. Bonferroni correction was used to minimized Type I error due to multiple testing (Bender and Lange, 2001). The cut-off *p*-value of 0.05 was divided by the number of tests performed. The new *p*-values for each test are reported in the “Results” section. Effect sizes were calculated using Cohen’s *d* and partial eta squared (η^2^*_*p*_*). For Cohen’s *d*, *d*-values < 0.50 were considered small, *d*-values between 0.50 and 0.80 were medium, and *d*-values above 0.80 were large. As for partial eta squared, we interpreted effect size values a follows: 0.01 ≤ η^2^*_*p*_* < 0.06 = small, 0.06 ≤ η^2^*_*p*_* < 0.14 = moderate, and 0.14 ≤ η^2^*_*p*_* = large (Cohen, 1988).

The M-Box test and Levene’s test were used to ensure homogeneity of group variance. The normal distribution of the data set was evaluated using the skewness and kurtosis values of each variable and Shapiro–Wilk’s test (Schumacker, 2016). The Cronbach Alpha (i.e. α) index was used to test the internal consistency of the scores in the questionnaires. Because the measurement scales of the questionnaires were ordinal, we corroborated the internal consistency values using Ordinal Alpha (*KR*-20), which is a more suitable internal consistency index for ordinal scales. Ordinal Alpha is interpreted in the same manner as Cronbach Alpha (Zumbo et al., 2007).

A latent variable, called Emotion Regulation, was created using LISREL 9.2 (Jöreskog and Sörbom, 2016). The scores on Maladaptive Emotion Regulation and Adaptive Emotion Regulation on the FEEL-KJ, the corresponding CERQ subscales, and the external regulation subscale of the FEEL-KJ were used to generate the latent variable. The Maximum Likelihood method was used for the analysis. The determinant of the covariance matrix was positive (0.44), indicating that there was variability among the variables after taking out the shared variance (Pituch and Stevens, 2016). The Kaiser-Meyer-Olkin (KMO = 0.50) test and the Bartlett test of sphericity [*X*^2^(10) = 198.06, *p* < 0.01] were run to determine whether the dataset was adequate to run this type of analysis (Schumacker and Lomax, 2010). For the KMO test, we accepted values above a recommended cut-off point of 0.50 (Tabachnick and Fidell, 2007) and for the Bartlett test a significance *p-*value of 0.05 was our cut-off point (Kaiser, 1974). Model fit statistics supported an adequate fit of our data to the specified model, χ^2^ = 4.00, *p* = 0.26, *df* = 3, NFI = 0.98, RMSEA = 0.03. The standardized solution weights ranged from −0.80 to 0.71.

To facilitate the interpretation of the generated Emotion Regulation latent scores in logits, we scaled the logit latent scores to a range from 0 to 100. For this purpose, we calculated a mean and standard deviation based on the logit scores and the new proposed scale (Bond and Fox, 2007). This mean and standard deviation were included in the formula *Mean+-Logit^∗^Standard Deviation* to transform the scores to the new scale (Allen and Schumacker, 1998). This transformation does not alter the characteristics of the dataset (Schumacker, 2006). The scaled Emotion Regulation scores were included in a univariate analysis to test main and interaction effects in relation to the children’s gender and age group (i.e. 9–12 years old or 13–16 years old).

## Results

The overall scores on FEEL-KJ and CERQ showed participant children and youth had higher scores in Adaptive Emotion Regulation strategies and lower scores in Maladaptive Emotion Regulation Strategies (Table 2). In general, the children and adolescents in the sample (9–16 years old) have good emotional regulation strategies evaluated at CERQ, with higher scores in Adaptive Emotion Regulation strategies (*Refocusing on planning*, the child or adolescent tries to think about how to solve the problem; followed by *Puting into perspective*, where the child or adolescent tries to lessen and relativize the severity of the situation) and lower scores on maladaptive strategies (*Other blame*, the child or adolescent holds others responsible for the negative event; followed by *Self blame*, the child or adolescent attributes the causes of the negative event and emotion to him or herself). Participants’ scores on the CERQ, ranged from 8.5 (Other-blame) to 13.56 (Refocus on planning). The overall mean CERQ score was 102.84, *SD* = 16.44.

The mean of the participants’ scores in the FEEL-KJ emotion subscales revealed higher scores in the anxiety subscale (*M* = 88.11, *SD* = 13.77) and lower scores in the anger subscale (*M* = 87.22, *SD* = 12.21), see Table 2. These results may indicate that the participants tend to use more emotion regulation strategies when experiencing anxiety than when experiencing sadness or anger. Nonetheless, the differences in the scores are less than a point, indicating that participants use emotion regulation strategies when experiencing sadness, anxiety or anger regardless.

As for the FEEL-KJ emotion regulation strategies, participant children and youth were more likely to use a problem solving strategy (*M* = 20.92, *SD* = 4.79) than other emotion regulation strategies and less likely to use aggressive action (*M* = 12.17, *SD* = 4.72) as an emotion regulation strategy (Table 2).

### Differences on the CERQ and FEEL-KJ Strategies by Gender

No statistically significant differences were identified for boys and girls when considering the nine CERQ strategies scores. The cut-off *p*-value for these tests was 0.005 after Bonferroni correction (Table 4). As for the FEEL-KJ strategies, girls scored statistically significantly higher than boys on Rumination (*p* = 0.001), Expression (*p* < 0.001), and Social Support (*p* = 0.001). The effect sizes of these differences were small (*d* = −0.47), moderate (*d* = −0.53), and small (*d* = −0.47), respectively. The cut-off *p*-value after Bonferroni correction for this set of analyses was 0.003. Table 4 shows the descriptive statistics, Student t-tests results, and effect size values (Table 4). The girls scored higher on emotional regulation by emotion and joint or grand mean scores. Statistically significant differences between boys and girls were observed in sadness-like emotions, anxiety. These results may indicate a greater use of regulation strategies by girls than by boys when experiencing sadness or anxiety (Table 5).

**TABLE 4:** Mean differences on the FEEL-KJ and CERQ emotion regulation strategies scores by gender and age group.

	Gender^a^					Age Group^b^				
Emotion regulation strategies	Girls	Boys	*tc*	*p*	*d*	9–12 years old	13–16 years old	*td*	*p*	*d*
**FEEL-KJ Strategies**										
Problem solving	21.06(4.58)	20.83(4.66)	–0.366	0.715	–0.050	20.87(5.13)	21.08(4.36)	–0.329	0.743	–0.044
Distraction	21.09(5.98)	21.06(6.52)	–0.028	0.978	–0.004	21.26(6.86)	20.76(5.77)	0.609	0.543	0.079
Forgetting	21.23(5.98)	20.50(6.29)	–0.864	0.389	–0.119	21.66(6.28)	20.17(6.01)	1.846	0.066	0.242
Acceptance	17.16(4.87)	17.60(4.28)	0.698	0.486	0.096	17.09(4.89)	17.62(4.21)	–0.873	0.383	–0.116
Humor Enhancement	20.30(4.80)	19.05(4.58)	–1.934	0.054	–0.266	19.68(5.00)	19.63(4.49)	0.083	0.934	0.010
Cognitive Problem Solving	21.49(4.57)	19.62(5.48)	–2.677	0.008	–0.369	20.33(5.37)	20.85(4.79)	–0.782	0.435	–0.102
Revaluation	16.20(4.87)	15.76(4.85)	–0.649	0.517	–0.089	15.44(5.21)	16.39(4.46)	–1.494	0.137	–0.196
Giving Up	15.07(4.74)	14.40(4.54)	–1.041	0.299	–0.143	14.6(4.82)	14.63(4.52)	–0.040	0.968	–0.006
Withdrawal	13.34(5.81)	12.97(5.37)	–0.481	0.631	–0.066	12.81(5.87)	13.57(5.23)	–1.031	0.303	–0.137
Rumination	19.54(4.66)	17.39(4.51)	–3.395	**0.001**	–0.468	17.92(4.29)	18.79(5.03)	–1.416	0.158	–0.187
Self-Devaluation	17.57(4.54)	17.05(4.23)	–0.866	0.387	–0.119	17.22(4.57)	17.52(4.21)	–0.524	0.601	–0.068
Aggressive Actions	13.09(4.98)	11.37(4.31)	–2.69	0.008	–0.371	11.94(4.82)	12.3(4.57)	–0.577	0.565	–0.077
Expression	16.43(6.68)	13.33(4.83)	–3.883	**<0.001**	–0.535	14.38(5.76)	15.25(6.12)	–1.118	0.265	–0.146
Social Support	20.10(6.88)	16.98(6.43)	–3.401	**0.001**	–0.469	18.19(6.32)	18.87(7.05)	–0.769	0.442	–0.101
Emotional Control	16.77(5.25)	17.30(5.41)	0.718	0.473	0.099	17.09(5.71)	17.05(4.92)	0.059	0.953	0.007
**CERQ Strategies**										
Self-Blame	9.55(3.30)	10.18(3.37)	1.369	0.172	0.192	9.08(3.44)	10.44(3.25)	–3.065	**0.002**	–0.406
Acceptance	11.28(3.403)	11.56(3.29)	0.593	0.554	0.083	10.77(3.39)	11.91(3.36)	–2.535	0.012	–0.337
Rumination	13.06(3.77)	12.07(3.57)	–1.926	0.056	–0.269	12.04(3.77)	12.83(3.65)	–1.588	0.114	–0.212
Positive Refocusing	12.10(4.92)	11.97(4.33)	–0.203	0.840	–0.028	13.08(4.59)	11.27(4.41)	3.039	**0.003**	0.402
Refocusing on Planning	14.20(3.83)	13.20(3.49)	–1.942	0.054	–0.272	12.97(3.69)	14.37(3.53)	–2.896	**0.004**	–0.387
Positive Reappraisal	12.44(3.84)	11.88(3.54)	–1.094	0.275	–0.153	11.91(3.73)	12.51(3.63)	–1.239	0.128	–0.163
Putting into Perspective	13.21(3.81)	12.69(3.60)	–0.989	0.324	–0.138	12.56(3.68)	13.10(3.62)	–1.115	0.217	–0.147
Catastrophizing	9.53(3.53)	10.13(3.56)	1.217	0.225	0.170	10.01(3.78)	9.70(3.27)	0.661	0.311	0.087
Other-Blame	8.19(3.29)	8.87(3.32)	1.481	0.140	0.207	8.58(3.59)	8.55(3.05)	0.058	0.026	0.009

*Bolded p-values highlight the stadistical significant results after the Bonfenrroni correction.^a^FEEL-KJ test (Girls n = 102; Boys n = 109); CERQ tests (Girls n = 108; Boys n = 97).^b^FEEL-KJ test (9–12 years old n = 118; 13–16 years old n = 115); CERQ tests (9–12 years old n = 118; 13–16 years old n = 109).^c^CERQ t-tests df = 225; FEEL-KJ t-tests df = 231.*

**TABLE 5:** Descriptive statistics and hotelling T^2^ test results for differences in the FEEL-KJ emotions Subscales Scores by participants’ gender.

FEEL-KJ Emotion	*M (SD)*	*F/t*	*df1*–*df2*	*p*	η_2_*p/d*
**Anger**					
Boys^a^	86.36 (11.63)	1,06	1–203	0.30	0.17
Girls^b^	88.48 (13.09)				
Boys and Girls^c^	87.40 (12.38)				
**Sadness**					
Boys^a^	84.85 (12.65)	8,56	1–203	<0.01	0.43
Girls^b^	90.63 (14.08)				
Boys and Girls^c^	87.67 (13.64)				
**Anxiety**					
Boys^a^	86.10 (12.84)	8,24	1–203	<0.01	0.41
Girls^b^	91.33 (12.78)				
Boys and Girls^c^	88.66 (13.04)				
**Joint or Grand Mean**					
Boys^a^	85.77 (12.37)	4.72	3–201	<0.01	0.07^d^
Girls^b^	90.15 (13.32)				
Boys and Girls^c^	87.91 (13.02)				

*^a^n = 108; ^b^n = 97; ^c^N = 205; ^d^η_2_p.*

### Differences on the CERQ and FEEL-KJ Strategies by Age Group

Regarding the nine CERQ strategies, older participants (13–16 years old) scored statistically significantly higher in Self-Blame (*p* = 0.002) and Refocus on Planning (*p* = 0.004) and lower in Positive Refocusing (*p* = 0.004) than younger participants (9 to 12 years old). Effect sizes were small for all three strategies, ranging from −0.39 to 0.40 (Table 4). No statistically significant differences were found among age groups when considering the fifteen FEEL-KJ strategies. The cut-off *p*-values after Bonferroni correction for differences in the CERQ (i.e., *p* = 0.005) and FEEL-KJ (i.e., *p* = 0.003) strategies scores by age group were the same as for the tests by gender. Table 4 shows the descriptive statistics and test results by age (Table 4).

Children ages 13 to 16 scored higher on the Sadness, Anxiety and Joint or Grand Mean emotion regulation scores. However, no statistically significant differences were seen in the use of emotional regulation strategies when considering emotion scores by age (Table 6).

**TABLE 6:** Descriptive statistics and hotelling T^2^ test results for differences in the FEEL-KJ emotions subscales scores by participants’ age group.

	*M (SD)*	*F/t*	*df1*–*df2*	*p*	η_2_*p*
**Anger**					
9 to 12 years old^a^	86.53 (12.40)	0.60	1–225	0.44	0.003
13 to 16 years old^b^	87.79 (11.89)				
Both age groups^c^	87.14 (12.15)				
**Sadness**					
9 to 12 years old^a^	87.78 (14.07)	0.01	1–225	0.97	0.000
13 to 16 years old^b^	87.83 (12.84)				
Both age groups^c^	87.81 (13.46)				
**Anxiety**					
9 to 12 years old^a^	87.69 (13.52)	0.54	1–225	0.46	0.002
13 to 16 years old^b^	88.97 (12.91)				
Both age groups^c^	88.3 (13.21)				
**Joint or Grand Mean**					
9 to 12 years old^a^	87.33 (13.33)	0.40	3–223	0.76	0.005
13 to 16 years old^b^	88.20 (12.55)				
Both age groups^c^	87.75 (12.94)				

*^a^n = 118; ^b^n = 109; ^c^N = 227.*

### Main and Interaction Effects of Gender and Age Group on Children’s and Adolescent’s Emotion Regulation Latent Scores

The variance analysis results revealed a main effect of age group on the emotion regulation latent scores, *F*(1, 201) = 4.11, *p* < 0.05, *n^2^_*p*_* = 0.02, *f*^2^ = 0.52. No main effects were found for participants’ gender. Nonetheless, an interaction effect of gender and age group on the children’s emotion regulation latent scores was supported, *F*(1, 201) = 15.47, *p* < 0.001, *n^2^_*p*_* = 0.07, *f*^2^ = 0.97. Younger girls tended to score higher than boys, and older girls scored lower than boys (see Table 7).

**TABLE 7:** Mean and standard deviations of the latent emotion regulation variable by age group and gender.

Age groups	*N*	*M (SD)*
**9 to 12 years old**		
Boys	59	55.33(17.17)
Girls	37	64.84(17.42)
Boys and Girls	96	58.99(17.8)
**13 to 16 years old**		
Boys	49	59.94(16.47)
Girls	60	50.41(16.99)
Boys and Girls	109	54.7(17.35)
**Total**		
Boys	108	57.42(16.94)
Girls	97	55.91(18.46)
Boys and Girls	205	56.71(17.65)

## Discussion

The objective of this study was to analyze the emotional regulation of children and adolescents from 9 to 16 years old in relation to age and gender. The results obtained with the FEEL-KJ and CERQ show that Adaptive Emotion Strategies were used to a greater extent than Maladaptive Emotion Regulation Strategies by the study participants. The characteristics of the instruments used have allowed this conclusion to be affirmed in a more convincing way since both tests distinguish between adaptive and maladaptive strategies. These results are to be expected since this is a non-clinical sample and is in line with different studies that analyze emotional regulation strategies in children and adolescents (John and Gross, 2004; Zimmer-Gembeck and Skinner, 2011).

Specifically, the results show that the strategies that present higher scores measured with the CERQ are Refocusing on planning and Putting into perspective, being these adaptive strategies, and those that present lower scores are Other blame and Self blame, which are maladaptive. These results are similar to those observed by Orgilés et al. (2018) in a Spanish sample aged 7–12 years, who found that the strategy most used by children and adolescents was *Refocusing on planning and* the less used *Other-blame*.

The results obtained with the FEEL-KJ show a higher use of Problem solving and Distraction (Adaptive Emotion Regulation Strategies) and a lower use of Agressive actions and Withdrawall (Maladaptive Emotion Regulation Strategies). These results are in line with previous studies that show that emotional regulation strategies considered as adaptive are used more frequently than strategies considered as maladaptive in adolescents (Garnefski et al., 2002).

In terms of gender differences, the FEEL-KJ results show that girls receive statistically significant higher scores on Sadness and Anxiety emotions, which may suggest a greater use of regulatory strategies when experiencing these emotions. However, since it’s outside the scope of this study, no analysis by emotion-based strategies has been conducted. Therefore, we cannot determine with certainty whether there is greater use of adaptive, maladaptive or external regulation strategies or the specific strategies that received the highest or lowest emotion-based scores. However, the results regarding differences between boys and girls generally indicate that girls express more positive and negative internalizing emotions than boys. This is in line with other research (Thoits, 1991, 1994; Tamres et al., 2002; Chaplin and Aldao, 2013).

Following the results obtained by the FEEL-KJ, we observe that girls have significantly higher scores than boys in the maladaptive strategy of Rumination and in the external regulation strategies of Expression and Social support. These differences are consistent with the results found in previous research (Tamres et al., 2002; Silk et al., 2003) and could be the result of contextual factors as suggested by Chaplin and Aldao (2013). However, there are no significant differences for the strategies reported by CERQ in terms of gender.

Analyzing the age differences in emotional regulation provided by the FEEL-KJ, children from 9 to 12 years old showed slightly lower scores than adolescents from 13 to 16 years old in emotional regulation strategies but the differences were not statistically significant. This is in line with research that indicates that the older the child, the greater the emotional regulation (Theurel and Gentaz, 2018). However, other studies such as Zimmermann and Iwanski (2014) point out that in middle adolescence (13–15 years), adolescents tend to present a decrease in strategies compared to early or late adolescence. This could be explained by the emotional difficulties and conflict with parents reported at this stage (Laursen et al., 1998). Analyzing the different strategies of emotional regulation presented by the FEEL-KJ, no significant results can be seen between the two age groups.

Regarding the age differences in emotional regulation shown in the CERQ, we observed that adolescents from 13 to 16 score significantly higher in the Self-blame and Refocusing on Planning strategies. Children and adolescents from 9 to 12 score statistically significant higher in the Positive Refocusing strategy. The Self-blame strategy is associated with a greater propensity to develop depressive and anxiety symptoms. On the other hand, the Refocusing on Planning and Positive Refocusing strategies may have a positive effect on the prevention of depression (Orgilés et al., 2018). These results support the idea mentioned above that during the stage of 13 to 16, adolescents would present more emotional problems (Zimmermann and Iwanski, 2014) since, as Chaplin and Aldao (2013) point out, maladaptive strategies such as Self-blame are strongly related to depressive symptoms, while adaptive strategies such as Refocusing on Planning are weakly related to such symptoms. Thus, proposals for intervention and prevention of psychopathology in this age group should focus on reducing or eliminating maladaptive strategies and strengthening and promoting the use of adaptive ones, since, as discussed above, evidence suggests that adaptive strategies can act as a protective factor (Orgilés et al., 2018).

Analyzing the previous results we see that to study the differences of emotional regulation according to gender we have obtained significant results through the FEEL-KJ but not through the CERQ and the opposite case to what happened when we wanted to study the differences of emotional regulation according to age. This may be interesting when considering future research on gender and age variables, since it seems that the FEEL-KJ could be more sensitive to detect strategies of emotional regulation according to gender and the CERQ according to age. However, this thesis will have to be investigated in greater depth to be able to affirm it with greater certainty. Furthermore, it is important to emphasize that in this study CERQ was used for participants over 12 years old and CERQ-Kids for those participants under 12 years old, which could indicate that the way questions are posed according to the developmental level of children or adolescents may have an impact on identifying differences in the use of emotional regulation strategies. As indicated in the introduction, the ability to modulate emotions is closely related to neurobiological maturation (Aldwin, 1994; Fields and Prinz, 1997; Stegge et al., 2004) a factor that should be considered when assessing emotional regulation strategies, as the authors of CERQ and CERQ-Kids do. Given that the FEEL-KJ covers a wide age range, and has not been adapted by age groups, this could explain why no differences by age group will be found with the FEEL-KJ. However, it is important to recognize that the authors of the FEEL-KJ consider the differences in relation to age when interpreting the results, dividing the scales by age group (Grob and Smolenski, 2005).

One of the strengths of our study is the creation of a latent variable, called *Emotion Regulation*, combining the participants’ scores on the factors of FEEL-KJ (adaptive, maladaptive and external regulation), a tool that combines internal and external strategies of emotional regulation, and the dimensions of CERQ (adaptive and maladaptive), which focus on internal regulation. The results show a more consistent agreement between the two scales in relation to the good fit of the model proposed to generate the latent variable.

When we analyzed the results of gender and age separately, we found significant results for gender when we used the FEEL-KJ, when we used CERQ we found significant results for age. However, when we use both scales to generate a latent variable, the differences by gender disappear and the statistically significant differences by age remain. As indicated by Chaplin and Aldao (2013), the factors that modulate gender differences in emotional regulation are age, interpersonal context and the type of task in which they are observed. This suggests that gender differences in the expression of emotions may not be static and fixed traits in individuals, but are mediated by complex interactions with the environment and with individual characteristics such as age. In this sense, the results found in this study, on the interaction between gender and age variables in relation to the scores of the latent emotional regulation variable, could support that gender differences in terms of emotional regulation are dynamic and could be explained by other variables such as age.

With regard with *latent emotional regulation factor scores’s* behavior, it was observed that girls obtain higher scores in emotional regulation at age 9–12 than boys and these scores decrease at age 13–16. This highlights the stage that is so vulnerable for girls at the emotional level at 13–16 years and which becomes evident with the increasing frequency in these ages of anxiety-depressive problems (Spence, 1998; Muris et al., 2000; Rodríguez de Kissack and Martínez-León, 2001; Orgilés et al., 2012).

Studies on emotional regulation tend to take into account age and gender separately, therefore, these results become more relevant since they focus on the interaction between gender and age, providing interesting information for a better understanding of emotional regulation in children and adolescents, thus allowing the design of more specific intervention programs.

### Limitations and Implications

The study has clear limitations that must be taken into account when interpreting and generalizing the results. One of the limitations of our study is given by the sample, future studies should have a random sample with a greater number of centers and subjects to ensure the representativeness of the results.

On the other hand, social desirability or poor introspection can alter the validity of results measured by self-reporting. This type of measurement may not be consistent with behavior in real situations. It would be interesting for future research to obtain information not only from children, but also from parents and teachers so that the information can be contrasted.

Likewise, the analysis of the emotional regulation strategies according to the FEEL-KJ emotions was done at the level of emotion (obtaining a general score of the use of strategies), the scores in the emotional regulation strategies according to each emotion (sadness, anger, anxiety) were not analyzed. Future studies could focus on this type of analysis to determine if by separating out emotion regulation strategies by emotion, differences in the use of certain strategies over others can be identified according to the emotion experienced. These differences could also be assessed in relation to the gender and age of the participants, to detect whether the differences found in this study are maintained when considering the choice of emotional regulation strategies based on the emotion being experienced.

Finally, as this is a transversal study, no causal relationships can be established, so it would be very interesting to carry out a longitudinal study.

As mentioned above, emotional regulation can be considered a transdiagnostic dimension. Since emotion regulation is a risk factor for the development of future psychopathology, techniques aimed at developing adaptive emotion regulation skills should be incorporated, both in treatment and in preventive interventions in childhood and adolescence. Our study highlights the adaptive and maladaptive strategies presented by children and adolescents and can indicate how to design prevention strategies and specific interventions according to gender and age.

## Data Availability Statement

The datasets generated for this study are available on request to the corresponding author.

## Ethics Statement

The studies involving human participants were reviewed and approved by the Comité de Ética de la Investigación, Universidad Católica de Valencia (IRB #: UCV-2017-2018-05). Written informed consent to participate in this study was provided by the participants’ legal guardian/next of kin.

## Author Contributions

All the authors have contributed jointly to the interpretation of the data as well as to the writing and review of the manuscript in order to obtain the final text.

## Conflict of Interest

The authors declare that the research was conducted in the absence of any commercial or financial relationships that could be construed as a potential conflict of interest.
